# In *Salmonella* Typhimurium and *Bacillus subtilis*, Nucleoid-Associated HU Proteins Are *N*-Terminally Acetylated

**DOI:** 10.3390/pathogens14070616

**Published:** 2025-06-20

**Authors:** Anastacia R. Parks, Jessica L. Will, Liju G. Mathew, Sébastien Massier, Julie Hardouin, Jorge C. Escalante-Semerena

**Affiliations:** 1 Department of Microbiology, University of Georgia, Athens, GA 30602, USA; anastacia_parks@hms.harvard.edu (A.R.P.); jesswill@uga.edu (J.L.W.); lijum9872@gmail.com (L.G.M.); 2 University of Rouen Normandie, INSA Rouen Normandie, CNRS, Polymers, Biopolymers, Surfaces Laboratory UMR 6270, F-76000 Rouen, France; sebastien.massier@univ-rouen.fr (S.M.); julie.hardouin@univ-rouen.fr (J.H.); 3 Université de Rouen Normandie, INSERM US 51 CNRS UAR 2026, HeRacLeS-PISSARO, Normandie Université, 76000 Rouen, France

**Keywords:** posttranslational modification, *N*-terminal acetylation, HU protein, Gram-negative bacteria, Gram-positive bacteria, protein acetylatransferase, *Salmonellla* pathogenesis

## Abstract

Here we report that the *Salmonella* Typhimurium NatB (*Se*NatB) protein *N*-terminal acetyltransferase acetylated the *N*-terminal methionine of the nucleoid-associated HU proteins. Our findings were supported by an in vitro analysis of acetylation of the HUα and HUβ proteins and lysine-null (K-null) variants, and by an in vivo analysis of the effect of acetylation on HU-mediated transcriptional regulation of a known target of HU, the *hilA* promoter. *Se*NatB did not acetylate the initiating methionines of HU proteins that were oxidized to methionine sulfoxide, but the reduction of these methionine sulfoxide residues restored the acetylation of HU proteins by *Se*NatB. These results demonstrate that the *Se*HU proteins are bona fide substrates for the methionine sulfoxide reductases MsrA and MsrB. Finally, we showed that the *Bacillus subtilis* acetyltransferase, YfmK, is a functional homolog of *Se*NatB, and that *Bs*YfmK acetylates the *N^α^* amino group of the initiating methionine of the *B. subtilis* HU protein (HBsu).

## 1. Introduction

Non-typhoidal *Salmonella enterica* subsp. *enterica* infections cause the diarrheal disease salmonellosis, which remains clinically significant due to its role as a leading cause of foodborne illnesses worldwide. It is estimated that global salmonellosis cases range from 93.8 to 153 million illnesses and 56,969 to 155,000 deaths per year, demonstrating a significant burden on public health and economic impact [1,2]. This bacterium employs a sophisticated array of virulence factors, including two Type III Secretion Systems (T3SSs) encoded by *Salmonella* Pathogenicity Islands 1 and 2 (SPI-1 and SPI-2), which facilitate the invasion of intestinal epithelial cells and survival within host macrophages, respectively [3,4]. Understanding these pathogenic mechanisms is crucial for developing targeted interventions, especially in the face of rising antimicrobial resistance among *Salmonella* strains [5]. Continued research into *S.* Typhimurium pathogenicity not only informs clinical management strategies but also aids in the development of effective vaccines and therapeutics to combat salmonellosis.

**Figure 1:**
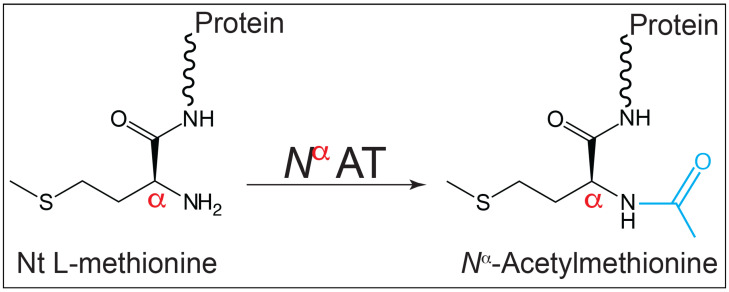
Schematic of the reaction catalyzed by an *N^α^* acetyltransferase (*N^α^*AT). The enzyme modifies the *α*-amino group of the initiating L-methionine yielding L-Met^Ac^ (iMet^Ac^).

*N*-terminal (*Nt*, also known as *N^α^*) acylation of the free amino moiety of the leading amino acid in a peptide chain (Figure 1) is a common occurrence in eukaryotic cells and serves a wide variety of purposes, such as targeting proteins for degradation [6,7,8,9], subcellular localization [10,11,12], protein folding [13,14,15], and protein–protein interactions [16,17,18,19,20,21].

In prokaryotes, however, knowledge of the extent and purpose of the *N^α^* acylation of proteins is limited. To identify these peptides in a large-scale study, peptide pre-fractionation methods followed by mass spectrometry analysis of all these fractions is a tedious but widely used method. Recently, several studies have taken an acylomics approach toward addressing the identity of *N*-terminally acylated proteins and acylation abundance in prokaryotes [22,23].

Here we took a targeted approach to advancing our understanding of the physiological impact of the *N^α^* acetylation of proteins in *Salmonella enterica* subspecies *enterica* serovar Typhimurium strain LT2 (hereafter *S.* Typhimurium). For this purpose, we used the *N^α^* acyltransferase (NatB) of this bacterium, which modulates the activity of the CobB sirtuin deacylase long isoform (hereafter CobB_L_) [24]. Our search for other protein substrates for *Se*NatB identified the homodimers of HU, HUα_2_ and HUβ_2_, and the hetero dimer HUαβ as substrates of *Se*NatB.

In *S.* Typhimurium, HU proteins are small (<10 kDa; 90 aa ea., Figure S1A) nucleoid-associated proteins (NAPs) that form three different types of oligomers, i.e., HUα_2_ or HUβ_2_ homodimers, or the HUαβ heterodimer. These relatively non-specific, DNA-binding proteins are present in *S.* Typhimurium cells in large amounts and influence the expression of many genes through changes in nucleoid compaction and organization [25]. The abundance of the different HU oligomers changes as a function of the growth phase of the cell [26], with the Lon protease involved in the degradation of the HUβ_2_ to allow a dimeric form exchange to take place [27].

Relevant to the work reported herein is the *Bacillus subtilis* GCN5-related acetyltransferase YfmK protein, which is known to acetylate epsilon amino groups of lysines in HBsu, the HU-like protein in *B. subtilis* [28], and here we show that YfmK can also catalyze the *N^α^* acetylation of HBsu. The degree of identity and similarity of HBsu to *S.* Typhimurium HU proteins is shown in Figure S1A.

Acetyltransferases possessing the GCN5-related N-acetyltransferase (GNAT) domain have a low sequence identity with each other, making the elucidation of homologs or their physiological roles difficult. Point in case, although an alignment of *Se*NatB and *Bs*YfmK shows only a 21.1% percent identity and 12% similarity between the two acetyltransferases (Figure S1B), evidence reported herein indicates that, like *Se*NatB, *Bs*YfmK has protein *N^α^* acetyltransferase activity, and that *Se*NatB and *Bs*YfmK N-terminally acetylate the HU proteins of *S.* Typhimurium and *B. subtilis*, respectively.

## 2. Materials and Methods

### 2.1. Bacterial Strains and Growth Conditions

All strains constructed were derivatives of either *Salmonella enterica* subsp. *enterica* sv Typhimurium str. LT2, *Salmonella enterica* subsp. *enterica* sv Typhimurium str. SB300, or *Salmonella* subsp. *enterica* sv Typhimurium str. ATCC 14028s. The reader is referred to Table 1 to identify the genetic backgrounds of the strains under discussion. Genes were deleted using described protocols [29]. Bacterial cultures were grown in lysogeny broth (LB) [30,31] at 37 °C with shaking unless stated otherwise.

### 2.2. Strain Construction

Deletions of the *hupA* and *hupB* genes were constructed as follows. Pfu Ultra II DNA polymerase (Stratagene) was used to amplify the flanking regions of the plasmid pKD3 [29] fused to 36 to 39 bp of internal to the beginning or end of the *hupA* or *hupB* genes. PCRs were resolved on a 1% (*w*/*v*) agarose gel and checked for amplification by post-staining with ethidium bromide (0.5 μg/mL) for 10 min. The PCR fragments were cleaned with the Wizard SV gel and PCR cleanup kit (Promega), and ~200 ng of product was electroporated into *S.* Typhimurium strain JE6692 (*metE205* Δ*araB9*/pKD46) using a 0.2 cm electroporation cuvette (MidiSci) and a microPulser electroporator (Bio-Rad Laboratories, San Francisco, CA USA) on Ec2 setting. Cells were incubated at 37 °C with shaking, plated on lysogeny broth (LB) supplemented with chloramphenicol (12.5 μg/mL), and incubated at 37 °C overnight. The insertion of *cat*^+^ was confirmed by PCR, then the marker and deletion were moved by P22-mediated transduction into strain JE6583 as described elsewhere [34]. Transductants were freed of phage, streaked under antibiotic selection and tested for phage sensitivity as described elsewhere [35]. The deletion of the gene was confirmed by Sanger sequencing using primers that flanked the gene of interest.

### 2.3. Plasmid Construction for Complementation and Overexpression

The plasmids used in this study are listed in Table 2. The plasmids were constructed as described elsewhere [24]. The K-null *Se*HUβ_2_ variant was constructed by the site-directed mutagenesis of *hupB* on plasmid pHUPB5. The primers used for this purpose were designed using Stratagene’s QuikChange Primer Design software package (https://www.agilent.com/store/primerDesignProgram.jsp, accessed on 16 June 2025).

PCR was conducted using Pfu Ultra II DNA polymerase (Agilent, Santa Clara, CA, USA) with primers listed in Table 3. Modification to this polymerase protocol included an extension temperature of 68 °C and an extension time of 2.5 min per kb. Mutations were confirmed by Sanger sequencing. The *Se*HUα_2_ K-null variant was synthesized by GenScript and cloned into pT7-7 using NdeI (5′) and XbaI (3′) restriction sites.

### 2.4. Purification of SeNatB and BsYfmK, SeHUα_2_, SeHUβ_2_, and SeHUαβ Proteins

#### 2.4.1. *Se*NatB and *Bs*YfmK

The plasmids pYIA6 and pYfmK1 were transformed into E. coli C41 (λDE3) Δpat (strain JE9314). Overnight cultures of *E. coli* that harbored either pYIA6 or pYFMK1 were sub-cultured 1:100 into 1 L each of Terrific Broth (TB; 12 g/L tryptone, 24 g/L yeast extract, 4 mL/L glycerol, MgCl_2_ (2 mM) buffered with KH_2_PO_4_ (0.17 M) plus K_2_HPO_4_ (0.72 M) containing ampicillin (100 μg/mL)). Cells were grown with shaking at 37 °C to an OD_600_ nm of 0.5, induced with isopropyl β-D-1-thiogalactopyranoside (IPTG, 0.5 mM), and then allowed to grow with shaking at 37 °C for 18 h. Filtered crude extracts were applied to a 1-mL HisBind FF column (GE Healthcare, Chicago, IL, USA) pre-equilibrated with Buffer A [2-[4-(2-hydroxyethyl)piperazin-1-yl]ethanesulfonic acid (HEPES) buffer (50 mM, pH 7.5) containing NaCl (500 mM), imidazole (20 mM), glycerol (5% *v*/*v*), and *tris*(2-carboxyethyl)phosphine hydrochloride (TCEP, 1 mM)] using an ÄKTA FPLC system (GE Healthcare). The column was washed with Buffer A and the proteins of interest were eluted with Buffer B [2-[4-(2-hydroxyethyl)piperazin-1-yl]ethanesulfonic acid (HEPES) buffer (50 mM, pH 7.5) containing NaCl (500 mM), imidazole (500 mM), glycerol (5% *v*/*v*), and *tris*(2-carboxyethyl)phosphine hydrochloride (TCEP, 1 mM)]. Fractions that contained each protein (as determined by SDS-PAGE) were pooled and dialyzed in Buffer A with 1 mM DTT for 3 h at room temperature with a 10 mg/1 mg ratio of MBP-6xHis-NatB or MBP-H_6_-YfmK to hexahistidine and *N*-terminally tagged recombinant tobacco etch virus (H_6_-rTEV) protease. Cleaved proteins were dialyzed twice more at 4 °C in Buffer A, then reapplied to a 1 mL HisBind FF column to separate the cleaved proteins from the MBP-H_6_ and H_6_-rTEV proteases. The cleaved proteins were collected in the flow through, analyzed by SDS-PAGE, pooled, and dialyzed in a storage buffer of HEPES (20 mM, pH 7.5) that contained NaCl (150 mM), TCEP (1 mM), and glycerol (20%, *v*/*v*).

#### 2.4.2. Native HU Heterodimer Purification

The α, β heterodimer of HU (HUαβ) was natively purified according to Pellegrini et. al. [40] as follows: overnight cultures of *Salmonella* strain JE6583 grown in lysogeny broth (LB) were sub-cultured 1:100 into 20 L of Terrific Broth (TB, tryptone (12 g/L) + yeast extract (24 g/L) + glycerol (4 mL/L) + MgCl_2_ (2 mM) buffered with KH_2_PO_4_ (0.17 M) and K_2_HPO_4_ (0.72 M) that contained ampicillin (100 μg/mL)). Cells were grown with shaking at 37 °C until the late-logarithmic phase when the HU heterodimer was most abundant compared with the homodimers [26]. The cells were pelleted at 6000× *g* for 15 min in a Beckman Coulter Avanti J-2 XPI centrifuge equipped with a JLA-8.1000 rotor (Beckman Coulter Life Sciences, Indianapolis, IN USA). The resulting 150 g cell pellet was resuspended in 900 mL of HU-purification Buffer A [Tris-HCl (20 mM, pH 7.8) that contained NaCl (1.7 M), ethylenediaminetetraacetic acid (EDTA, 1 mM), and 2-mercaptoethanol (1 mM)] and was lysed by passage through a TS-Series 0.75 kW cell disruptor (Constant Systems, Daventry, UK) at 120 and 138 MPa. The cell debris was then pelleted for 30 min at 6000× *g* in the centrifuge listed above and the cleared supernatant was saved. Polyethylene Glycol (PEG, 4000 Da) was added to the cell-free extract at a final concentration of 15% (*w*/*v*) and was mixed slowly for 2 h at 4 °C. The cell-free extract was centrifuged again for 30 min at 12,000× *g*. The supernatant after centrifugation was dialyzed over the span of 24 h thrice, each time against 2 L of HU-purification Buffer B [Tris-HCl (20 mM, pH 7.8) containing NaCl (200 mM) + EDTA (1 mM)]. The dialyzed cell-free extract (dCFE) was centrifuged for 20 min at 12,000× *g*, then applied to a 20 mL HiPrep Heparin FF 16/10 column attached to an ÄKTA FPLC system, with 150–200 mL of dCFE applied per run for a total of three runs. A linear elution gradient from 0.2 to 1 M NaCl in Buffer B was applied; the HUαβ heterodimer was eluted at approximately 0.65 M NaCl. The HiPrep Heparin FF 16/10 column was cleaned in between runs with 80 mL of NaOH (0.1 M). The eluted HU fractions were pooled and dialyzed twice against 1 L of Tris-HCl (20 mM, pH 7.8) that contained NaCl (50 mM) and EDTA (1 mM), then the HU isoforms were separated by running the dialyzed protein over 5 mL of P11 phosphocellulose resin via gravity. The HU isoforms were eluted from the resin with the application of a linear gradient of 0.05 to 1 M NaCl, and 10 mL fractions were collected in 50 mM increments of NaCl; the HUαβ heterodimer was eluted at between 0.45 and 0.5 M NaCl.

To ensure the HU isoforms were separated, fractions were resolved by 7.5% Triton-X100 acid-urea (TAU) gels, an acidic gel system used to separate HU isoforms based on their pI. The gel recipe for TAU was as follows: glacial acetic acid (25%, *v*/*v*), urea (2.5 M), Triton-X100 (1%, *w*/*v*), riboflavin (14 mM), bis-acrylamide (0.2 M), and ammonium persulfate (APS, 8.25 mM) + N,N,N′,N′-tetramethylethylenediamine (TEMED, 5.4 mM) per 8 mL gel volume. The gels were solidified by exposure to UV for 30 min. The samples of fractions from the phosphocellulose column were suspended in TAU sample buffer (acetic acid (5% (*v*/*v*), glycerol (50%, *v*/*v*) plus methyl green (13 mM), then resolved by TAU electrophoresis with an acetic acid (5% (*v*/*v*), pH 3.6) running buffer at 200 V for 45 min at room temperature with 5 μL of 10 mg/mL cytochrome C used as a running marker. The anode and cathode leads were reversed to ensure electrophoresis to the negative cathode. Fractions that contained the heterodimer of HU as verified by TAU electrophoresis were pooled and mixed by a slow addition of ammonium sulfate to a final concentration of 2.7 M. The ammonium sulfate–protein mixture was applied to a 5 mL Phenyl Sepharose FF column on the FPL chromatograph and an inverse linear gradient from 2.7 M to 0 M ammonium sulfate was performed to elute the heterodimer HU. The fractions with elution peaks that corresponded to the FPL chromatogram were dialyzed in a HEPES buffer (50 mM, pH 7.5) that contained NaCl (50 mM) and glycerol (10%, *v*/*v*) to verify the presence of HU proteins by 15% SDS-PAGE [41]. Once the fractions were confirmed, they were concentrated using a Centricon Plus-70 centrifugal filter with a 3 kDa cutoff (Millipore, Burlington, MA, USA) as per manufacturer instructions and flash frozen in liquid nitrogen in storage buffer and stored at −80 °C until used.

#### 2.4.3. Plasmid-Derived *Se*HUα_2_, *Se*HUβ_2_, and Lysine Variants Purification

The homodimers and lysine variants of the homodimers were purified as described above, except as follows: Overnight cultures of *S.* Typhimurium strain JE13152 (Table 1) harboring an arabinose-inducible, chromosomally-encoded T7 RNA polymerase and either plasmid pHBSU1, pHUPB5, pHUPB14, pHUPB15, pHUPA3, pHUPA6, or pHUPA7 were grown in LB, then sub-cultured 1:100 into 1.5 L of TB supplemented with L(+)-histidine (5 mM) and grown with shaking at 37 °C to an optical density at 600 nm (OD_600_) of 0.7–0.8. T7 RNA polymerase synthesis was induced by the addition of L-(+)-arabinose to the culture and the plasmid encoding of the HU protein of interest was induced by the addition of IPTG (0.5 mM) to the medium. Gene expression was induced overnight, and cells were harvested the next morning by centrifugation at 6000× *g* for 15 min. The protein purification protocol used was the same as that outlined above under Native HU Heterodimer Purification and as described in [40].

#### 2.4.4. Purification of *Se*MsrA

MsrA cloned into pTEV18 rTEV-cleavable N-terminal H_6_-fusion was electroporated into *E. coli* C43 (λDE3) cells and plated onto LB agar that contained ampicillin (100 μg/mL). The next day, a single colony was inoculated into 10 mL of LB containing ampicillin (100 μg/mL) and grown overnight with shaking at 37 °C. The next morning, 1 L of LB was inoculated with the 10 mL culture and grown to an OD_600_ of 0.6. The plasmid was induced with IPTG (1 mM) and grown with shaking at 37 °C overnight. The cells were centrifuged as described above and the resulting cell pellet was resuspended in a Tris-HCl buffer (50 mM, pH 8) that contained NaCl (0.5 M), imidazole (0.02 M), dithiothreitol (DTT, 0.02 M), phenylmethanesulfonyl fluoride (PMSF, 1 mM), lysozyme (1 mg/mL), and DNase I (1 μg/mL). The resuspended cells were sonicated 3× for 60 s total, with 2 s on and 2 s off at a 60% amplitude on a Qsonica sonicator. The lysate was centrifuged at 30,000× *g* for 30 min in a Beckman Coulter Avanti J-25I centrifuge equipped with a JA-25.50 rotor centrifuge (Beckman Coulter, Indianapolis, IN, USA). The resulting supernatant that contained soluble H_6_-MsrA was purified by gravity column chromatography on a 1 mL HisPur Ni-NTA affinity chromatography resin. The resin was washed with 10 mL of Tris-HCl (50 mM, pH 8) that contained NaCl (0.5 M), imidazole (0.02 M), and DTT (0.02 M), followed by a 7 mL wash using the previous buffer mixed with 4% (*v*/*v*) of Tris-HCl (50 mM, pH 8) that contained NaCl (0.5 M), imidazole (0.5 M), and DTT (0.02 M). H_6_-MsrA protein was eluted off the column with 1 mL of a Tris-HCl buffer (50 mM, pH 8) that contained NaCl (0.5 M), imidazole (0.5 M), and DTT (0.02 M). H_6_-MsrA protein was dialyzed thrice at 4 °C against a Tris-HCl buffer (50 mM, pH 8) that contained glycerol (20%, *v*/*v*), NaCl (0.1 M), and DTT (0.02 M), and then flash frozen at −80 °C until used.

#### 2.4.5. Purification of *Se*MsrB

The *S.* Typhimurium *msrB* gene was cloned into cloning vector pTEV18 [36]. The resulting plasmid directed the synthesis of a MsrB protein with a recombinant tobacco etch virus (rTEV) protease-cleavable, N-terminal H_6_-fusion MsrB protein (hereafter H_6_-*Se*MsrB). The resulting plasmid is hereafter referred to as pMsrB1. pMsrB1 was electroporated into *E. coli* C43 (λDE3) cells and plated onto an LB agar that contained ampicillin. The next day, a single colony was inoculated into 10 mL of LB containing ampicillin (100 μg/mL) and grown overnight with shaking at 37 °C. The next morning, 1 L of LB was inoculated with the 10 mL overnight culture and grown to an OD_600_ of 0.6. The plasmid was induced with IPTG (1 mM) and grown for an additional 4 h. The cells were centrifuged as described above and the resulting cell pellet was resuspended in a Tris-HCl buffer (50 mM, pH 8) that contained DTT (0.02 M), PMSF (1 mM), lysozyme (1 mg/mL), and DNase I (1 μg/mL). The resuspended cells were sonicated 3× for 60 s total, with 2 s on and 2 s off at a 60% amplitude on a Qsonica sonicator (Sonics & Materials, Inc., Newton, CT, USA). The lysate was centrifuged at 30,000× *g* for 30 min in a Beckman Coulter Avanti J-25I centrifuge equipped with a JA-25.50 Beckman Coulter rotor. The overexpressed H_6_-*Se*MsrB protein was found in the insoluble pellet, so the supernatant was removed, and the insoluble pellet was resuspended in a Tris-HCl buffer (50 mM, pH 8) that contained imidazole (0.02 M) and urea (6 M). A 1 mL slurry of HisPur Ni-NTA resin was used to purify the H_6_-*Se*MsrB protein by gravity. The insoluble cell lysate was applied to the resin, followed by washing the resin with 10 mL of a Tris-HCl buffer (50 mM, pH 8) that contained imidazole (0.02 M) and urea (6 M). A second wash was performed with 7 mL Tris-HCl buffer (50 mM, pH 8), imidazole (0.04 M), and urea (6 M). The H_6_-MsrB protein was eluted with 1 mL of a Tris-HCl buffer (50 mM, pH 8) that contained imidazole (500 mM) and urea (6 M). The eluted H_6_-MsrB protein was slowly refolded by dialysis at 4 °C and a stepwise decrease in the urea concentration in a Tris-HCl buffer (50 mM, pH 8) that started with 4 M urea, then decreased to 3 M, 1.5 M, 0.5 M, and lastly no urea. Each dialysis was performed against a one-liter volume of buffer and the protein was dialyzed for 3 h in each buffer at 4 °C. The resulting refolded H_6_-MsrB protein was filtered through a 0.45 mm filter and flash frozen at −80 °C in a Tris-HCl buffer (50 mM, pH 8) that contained glycerol (20%, *v*/*v*).

### 2.5. Thin-Layer Chromatography (TLC) Assay of Msr Activity

Msr activity assay reactions were set up in 50 μL volumes that contained 10 μg of MsrA and/or MsrB proteins incubated with 10 μL of 100 mM DTT, 2.5 μL of HEPES buffer (1 M, pH 7), and 5 μL of 100 mM methionine sulfoxide (Met-SO). Reactions were incubated at 37 °C for 2 h, then 10 μL of each reaction was spotted in 1 μL increments onto a Baker flex silica gel IB2-F TLC plate with a one-half centimeter spacing and 1 cm wide lanes. Meanwhile, a TLC chamber was equilibrated with N-butanol:acetic acid/water at a 60:12:25 ratio for several hours. The dried TLC plate was developed in the chamber until the mobile phase reached close to the top of the plate. The plate was dried and sprayed with a ninhydrin spray that contained 0.3 g of ninhydrin in a mixture of N-butanol (100 mL) with acetic acid (3 mL). The plate was left to dry for less than 10 min at 70 °C to develop.

### 2.6. Mass Spectrometry Methods and Data Analysis

#### 2.6.1. At the University of Georgia: Asp-N Digestion and LC-MS/MS Analysis

HU proteins were resolved on a 7.5% TAU gel as described above and stained/destained with Coomassie and acetic acid to visualize the proteins. Protein bands of interest were excised from the gel, and the in-gel digestion of the HU proteins was conducted according to the Promega Asp-N sequencing grade In-Gel digestion protocol (cat# V162A). The digested samples were cleaned up using C18 solid phase extraction (SPE C18), and an LC-MS/MS analysis was conducted on Orbitrap Elite systems.

The enzymatic peptides were loaded into a reverse-phase column (self-packed column/emitter, 0.1× ~150 mm ID, with 200 Å 5 µM Bruker MagicAQ C18 resin, Billerica, MA, USA), then directly eluted into the mass spectrometer at a flow rate of 450 nL/min. Briefly, the two-buffer gradient elution (0.1% formic acid as Buffer A and 99.9% acetonitrile with 0.1% formic acid as Buffer B) started with 0% B; was held at 0% B for 2 min; and then increased to 12% B in 25 min, to 30% B in 25 min, to 50% B in 10 min, and to 95% B in 10 min. The data-dependent acquisition (DDA) method was used to acquire MS data. A list of expected precursor ions was generated for monitoring the possible *N*-terminal peptides with one missed cleavage. A survey MS scan was acquired first (*m*/*z* 350–1500), and then the top four ions in the precursor list were selected for CID followed by HCD MS/MS analysis with an isolation width of 2 *m*/*z*. If the no-peptide ion in the precursor list was found, the most abundant ions were chosen for MS/MS analysis. Both MS and MS/MS scans were acquired by Orbitrap at resolutions of 120,000 and 15,000, respectively. Data were acquired using Xcalibur software (version 2.2, Thermo Fisher Scientific, Waltham MA, USA). Protein identification and modification characterization were performed using Thermo Proteome Discoverer (version 1.4) with Mascot (Matrix Science, London, UK) and Uniprot *Salmonella* database. The search parameters included the following: (i) precursor mass tolerance: 10 ppm; (ii) fragment mass tolerance: 0.02 Da; (iii) modification: oxidation of Methionine; and (iv) validated with percolator (decoy database), targeted FDR (restrict/relax): 0.01/0.05. The extracted ion chromatograms (EICs) of N-terminal peptides were plotted by combining various peptide ions at various charges within the mass tolerance of 5 ppm of MS1. ICIS peak detection in Xcalibur is the default method to integrate the peak area. P.S.: 1 Thomson = 1 *m*/*z*.

#### 2.6.2. At the University of Wisconsin-Madison: Enzymatic “In Gel” Digestion

“In gel” digestion and mass spectrometric analysis was performed at the Mass Spectrometry Facility [Biotechnology Center, University of Wisconsin-Madison]. In short, excised gel pieces were washed twice for 2 min in MeOH:H_2_0:NH_4_HCO_3_ [50%:50%:100 mM], dehydrated for 2 min in ACN:H_2_0:NH_4_HCO_3_ [50%:50%:25 mM], then once more for 30 s in 100% ACN, dried in a Speed-Vac for 1 min, rehydrated completely and reduced in 25 mM DTT [Dithiotreitol in 25 mM NH_4_HCO_3_] for 30 min at 56 °C, alkylated by solution exchange with 55 mM IAA [Iodoacetamide in 25mM NH_4_HCO_3_] in darkness at room temperature for 30 min, washed once in 25 mM NH_4_HCO_3_, dehydrated twice for 2 min in ACN:H_2_0:NH_4_HCO_3_ [50%:50%:25 mM], then once more for 30 s in 100% ACN, dried in a Speed-Vac again, and finally rehydrated with 20 μL of trypsin solution [10 ng/μL trypsin (PROMEGA) in 25 mM NH_4_HCO_3_ and 0.01% ProteaseMAX *w*/*v* (PROMEGA)]. An additional 30 μL of digestion solution [25 mM NH_4_HCO_3_ and 0.01% ProteaseMAX *w*/*v* (PROMEGA)] was added to facilitate the complete rehydration with excess overlay needed for peptide extraction. The digestion was conducted for 3 h at 42 °C. Peptides generated from digestion were transferred to a new tube and acidified with 2.5% TFA [Trifluoroacetic Acid] to 0.3% final. The degraded ProteaseMAX was removed via centrifugation [max speed, 10 min] and the peptide solid phase was extracted (ZipTip^®^ C18 pipette tips Millipore, Billerica, MA, USA).

##### NanoLC-MS/MS

The peptides were analyzed by nanoLC-MS/MS using the Agilent 1100 nanoflow system (Agilent, Santa Clara, CA, USA) connected to a hybrid linear ion trap–orbitrap mass spectrometer (LTQ-Orbitrap Elite™, Thermo Fisher Scientific) equipped with an EASY-Spray™ electrospray source. The chromatography of the peptides prior to the mass spectral analysis was accomplished using a capillary emitter column (PepMap^®^ C18, 3 µM, 100 Å, 150 × 0.075 mm, Thermo Fisher Scientific) onto which 2 µL of extracted peptides was automatically loaded. The NanoHPLC system delivered solvents A: 0.1% (*v*/*v*) formic acid and B: 99.9% (*v*/*v*) acetonitrile and 0.1% (*v*/*v*) formic acid at 0.50 µL/min to load the peptides (over a 30 min period) and 0.3 µL/min to elute the peptides directly into the nano-electrospray with a gradual gradient from 3% (*v*/*v*) B to 20% (*v*/*v*) B over 17 min, followed by a 5 min fast gradient from 20% (*v*/*v*) B to 50% (*v*/*v*) B, and concluded with a 4 min ramp to 95% (*v*/*v*) B, at which time a 1 min flush-out took place. As the peptides eluted from the HPLC-column/electrospray source survey MS scans were acquired in the Orbitrap with a resolution of 120,000 followed by MS2 fragmentation of the 20 most intense peptides detected in the MS1 scan from 350 to 1800 *m*/*z*, redundancy was limited by dynamic exclusion.

##### Data Analysis

Raw MS/MS data was converted to the mgf file format using MSConvert (ProteoWizard: Open Source Software for Rapid Proteomics Tools Development v. 3.0). The resulting mgf files were used to search against a user-defined amino acid sequence database containing *Salmonella enterica* CobB sequences (long and short variants), along with a list of common contaminants using the in-house *Mascot* search engine 2.2.07 [Matrix Science] with variable Lysine and protein N-terminus acetylation, Methionine oxidation, Asparagine, and Glutamine deamidation plus fixed Cysteine carbamidomethylation. The peptide mass tolerance was set at 15 ppm and the fragment mass at 0.6 Da. All the significant identifications based on the ion scores were manually interrogated to confirm the algorithmic assignments. The extracted ion chromatograms were generated to evaluate the abundances between samples and raw MS2 files inspected for the correctness of identification.

### 2.7. In Vitro Acetylation Assays

Homogeneous HU proteins (5 mM) were incubated with [1-^14^C]-AcCoA (40 mM) in a HEPES buffer (50 mM, pH 7) that contained TCEP (1 mM) with or without *Se*NatB protein (3 mM) for 1 h at 37 °C in a total volume of 25 μL. The reactions were stopped by the addition of 5 μL of SDS-loading dye (glycerol (60%, *v*/*v*), Tris-HCl buffer [0.3 M, pH 6.8], ethylenediaminotetracetic acid (EDTA, 12 mM), sodium dodecyl sulfate (SDS, 12% *w*/*v*) plus 2-mercaptoethanol (0.87 mM), and bromophenol blue (0.05%, *w*/*v*). The samples were resolved by SDS-PAGE at 200 V for 45 min in 20% (*w*/*v*) polyacrylamide gels with Tris-HCl buffer pH 8.8 as the resolving buffer in the gel, and Tris-HCl buffer pH 6.8 as a stacking layer buffer in the gel. The gels were stained with Coomassie dye [Brilliant Blue R (1 g/L), isopropanol (25%, *v*/*v*), glacial acetic acid (10%, *v*/*v*)] and exposed for two days to BAS storage phosphor screens (BAS-IP MS 2040 E, GE Healthcare, Chicago, IL, USA). Radiolabel transfer was visualized by imaging on the phosphor imaging setting using a Typhoon trio Plus variable mode imager (GE Healthcare).

### 2.8. In Vitro Deacetylation Assays

Deacetylation assays were conducted by first setting up in vitro acetylation assays as described above. Acetylation reactions were then buffer exchanged with an Amicon Ultra 0.5 mL centrifugal filter Ultracel-3K (3 kDa molecular mass cutoff) to remove the excess AcCoA. Proteins bound to the Amicon filter were washed with 400 μL of HEPES buffer (50 mM, pH 7). Eluted HU proteins or Acs protein (used as a positive control) were brought to a concentration of 3 μM in the HEPES buffer (50 mM, pH 7). Each eluted HU or Acs protein was present at a final concentration of 3 μM in a reaction mixture that contained dithiothreitol (DTT, 2mM), NAD^+^ (2 mM), CobB_S_ protein (2 mM), and HEPES buffer (50 mM, pH 7). Negative controls included a reaction without CobB_S_ protein added. The reaction mixtures were incubated at 37 °C for one hour, terminated by the addition of SDS loading dye, and resolved and visualized as described under ‘In vitro acetylation assays’.

### 2.9. Galactosidase Assays

The method described by Miller was used to quantify the level of β-galactosidase enzyme activity [42]. Two biological replicates of each strain were grown overnight in LB plus ampicillin (100 μg/mL). In the morning, the cells were sub-cultured 1:100 (*v*/*v*) into 4 mL of LB that contained ampicillin in 10 × 75 mm borosilicate glass tubes with tight-fitting lids. L-(+)-arabinose (0.5 mM) was added to induce complementation plasmids. The cultures were placed in a 37 °C incubator and left static until the cells had reached an OD_600_ of 0.5–0.6. Miller units were calculated using the Miller equation: 1000 × [(OD_420_ − (1.75 × OD_550_)/(T × V × OD_650_)], where T is the reaction time in minutes, and V is the volume of culture used in ml. The Miller units were plotted and the statistical analysis was performed in Prism 6 software (GraphPad, Boston, MA, USA).

### 2.10. Electrophoretic Mobility Shift Assays (EMSAs)

5(6)-Carboxyfluorescein [5(6)-FAM]-labeled DNA oligonucleotides were purchased from Integrated DNA Technologies (IDT, Coralville, IA, USA). dsDNA probes were generated by PCR (polymerase chain reaction) using PFU Ultra II polymerase (Agilent) according to the manufacturer’s protocol. The DNA probes were cleaned of excess labeled nucleotides using a Promega PCR and Gel Clean up Kit. A total of 50 ng of *hilA* promoter DNA was used per reaction in a total reaction volume of 25 μL. The EMSA buffer contained a final concentration of Tris-HCl buffer (10 mM, pH 7.6) that contained NaCl (15 mM), glycerol (15% (*v*/*v*)), and EDTA (0.1 mM). Increasing concentrations of *Se*HUα_2_ protein were added in excess to the DNA probe. The reactions were incubated at room temperature for 10 min, then 5 μL of glycerol (50%, *v*/*v*) was added to each reaction and 20 μL of each reaction was resolved by native polyacrylamide gel electrophoresis using a non-denaturing Criterion Tris-HCl buffer (375 mM, pH 8.6) that contained polyacrylamide (7.5% (*w*/*v*)) (Bio-Rad Laboratories, Hercules, CA, USA) gels in a Tris-Borate-EDTA (0.5× TBE) buffer; the samples were resolved at 120 V. An empty lane was loaded with 2 mL of 10× DNA loading dye (which contained 50% glycerol, bromophenol blue, and xylene cyanol) and was used as a visual tracking aid. The gels were electrophoresed at 4 °C until the bromophenol blue indicator reached the bottom of the gels. The gels were imaged using a Typhoon Trio^+^ variable imager (GE Healthcare) at a wavelength of 488 nm and quantified with ImageQuant v5.2 software. Densitometry measurements were made using ImageJ software v 0.5.8, where the pixel intensity was measured, background pixels were subtracted, and the ratios of bound DNA bands to unbound DNA bands were calculated for each EMSA lane. The ratios were calculated in Microsoft Excel, and the ratios were plotted using Prism 10 software (GraphPad).

## 3. Results

### 3.1. Nomenclature Clarification

In a previous publication from our laboratory [24], we suggested changing the *yiaC* name (gene encoding a protein of unknown function) to *natA* (a gene encoding an *N^α^*-acetyltransferase). Unfortunately, the suggested name did not consider the established substrate specificity differences between NatA and NatB enzymes [43,44]. To be consistent with the literature and avoid confusing the reader, we have corrected the name of YiaC to be NatB.

### 3.2. In S. Typhimurium, SeNatB Acetylated All Three Forms of HU

**Figure 2:**
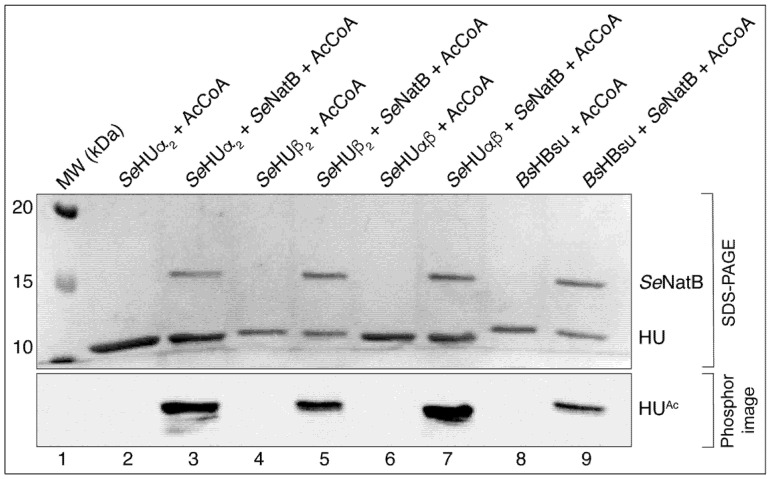
*Se*NatB acetylated *Se*HUβ_2_, *Se*HUα_2_, *Se*HUαβ_2_, and *HBsu* in vitro. The ability of *Se*NatB to acetylate the HU proteins was assessed after the incubation of 40 μM [acetyl-1-^14^C]-AcCoA with *Se*NatB (2 μM) and either *Se*HUα_2_, *Se*HUβ_2_, *Se*HUαβ, and *HBsu* proteins (5 μM each) for one hour at 37 °C. The bands seen on the phosphor image indicate acetylated protein that corresponds to protein bands from SDS-PAGE seen at the size of HU proteins, namely, 10 kDa. The controls included reaction mixtures that contained each of the HU proteins plus [acetyl-1-^14^C]-AcCoA, but no acetyltransferase. The proteins were resolved by SDS-PAGE and visualized by Coomassie Blue R staining (**top image**) using Precision Plus protein standard (Bio-Rad Laboratories) as the molecular weight (MW) marker. The distribution of the radiolabel signal was visualized by phosphor imaging (**bottom image**). This experiment was repeated three independent times.

During the screening experiments for potential substrates for putative GCN5-related *N*-acetyltransferases (i.e., GNATs), we found that the *S. Typhimurium* NatB (*Se*NatB) protein acetylated the HU proteins of this bacterium. As seen in Figure 2, *Se*NatB efficiently acetylated the homodimeric *Se*HUα_2_ (Figure 2, lane 3), homodimeric *Se*HUβ_2_ (Figure 2, lane 5), and heterodimeric *Se*HUαβ (Figure 2, lane 7). We also found that *Se*NatB acetylated the *B. subtilis* homolog of HU, namely, HBsu (Figure 2, lane 9).

**Figure 3:**
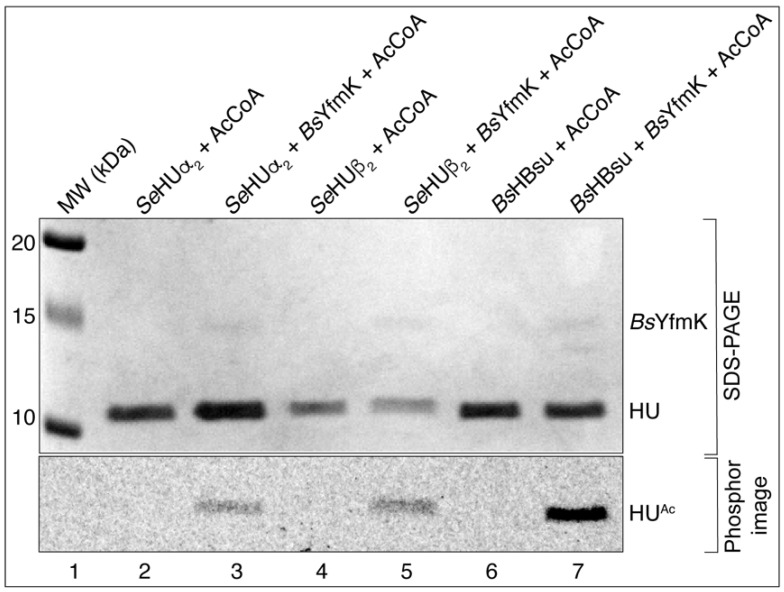
*Bs*YfmK could acetylate the *Se*HU and HBsu proteins. The ability of *Bs*YfmK to acetylate the HU proteins was assessed after the incubation of 40 μM [acetyl-1-^14^C]-AcCoA with *Bs*YfmK (2 μM) and either a *Se*HUβ_2_, *Se*HUα_2_, or HBsu protein (5 μM each) for one hour at 37 °C. The bands seen on the phosphor image indicate acetylated the protein that corresponded to protein bands from the SDS-PAGE seen at the size of the HU proteins, namely, 10 kDa. The controls included reaction mixtures that contained each of the HU proteins plus [acetyl-1-^14^C]-AcCoA, but no acetyltransferase. The proteins were resolved by SDS-PAGE and visualized by Coomassie Blue R staining (**top image**) using Precision Plus protein standard (Bio-Rad Laboratories) as the molecular weight (MW) marker. The distribution of the radiolabel signal was visualized by phosphor imaging (**bottom image**). This experiment was repeated three independent times.

This result prompted us to test whether *B. subtilis* YfmK (hereafter *Bs*YfmK) could acetylate the *Se*HU isoforms because of the high sequence identity (~50%) and ~10% similarity between the *Se*HU and HBsu proteins (Figure S1A). As shown in Figure 3, *Bs*YfmK acetylated the HBsu, *Se*HUα_2_, and *Se*HUβ_2_ proteins. The intensity of acetylation of *Se*HU proteins by *Bs*YfmK was lower than *Bs*YfmK for HBsu. It is unclear whether this was due to the *Se*HU proteins being poor substrates for *Bs*YfmK. We purified the *Se*HUαβ protein natively from 20 L of culture of a *S. Typhimurium* LT2 strain that harbored no plasmids using a described protocol [40]. From these cells we separated the three biological isoforms of *Se*HU. To isolate the homogeneous *Se*HUα_2_, *Se*HUβ_2_, or HBsu protein, a *hupA hupB* deletion strain of *S.* Typhimurium that harbored a chromosomally encoded T7 polymerase was complemented with a T7-7 overexpression vector that encoded either *hupA*, *hupB*, or the *B. subtilis hbs* gene (the gene encoding HBsu, equivalent to *Se*HupA). Our results are described below.

### 3.3. Several SeHU Variant Proteins Existed In Vivo

The primary sequence of the *Se*HUα contained nine lysyl and three arginyl residues, whilst the *Se*HUβ protein contained nine lysyl and five arginyl residues (Figure S1A). This content of lysyl and arginyl residues complicated the LC-MS/MS analyses when the proteins were digested with trypsin because trypsin cleaves the peptide bond’s C-terminal to lysine and arginine. To circumvent this problem, we used endoproteinase Asp-N to determine where *Se*NatB modified the homodimers of the *Se*HU proteins. Endoproteinase Asp-N is known to cleave the peptide bond’s N-terminal to aspartic or cysteic acid residues, and in the case of AspN-treated HU proteins, we obtained peptides long enough for analysis using LC-MS/MS [45,46] (Figure S2). To obtain a baseline of *N*-terminal peptide coverage, we digested native *Se*HUα and *Se*HUβ proteins with Asp-N, and the resulting peptides were analyzed by LC-MS/MS. From these experiments we did not detect *N-*acetylated peptides, but we discovered several sub-populations of *Se*HU proteins (Figures S3 and S4): (i) a sub-population that lacked the initiating methionine (iMet), (ii) another sub-population that had the iMet residue but it had been oxidized to methionine sulfoxide, and (iii) a third sub-population that had the iMet. These data indicate that for this protein population, the formyl group of the initiation formyl-methionine was removed by peptide deformylase to generate free iMet, and potentially a sub-population of these HU proteins had their iMet removed in vivo. The population of oxidized Met residues was not surprising since methionine oxidation is common in cells undergoing oxidation stress, hence these oxidized proteins were likely part of the population we isolated from the cells [47]. Using the calculated area under the curve compared with the total ion counts (TICs), we quantified the sub-population abundance, i.e.*,* the total of each population divided by the total population of peptides. We noticed that the alluded sub-populations were similar between the *Se*HUα and *Se*HUβ proteins. The *Se*HUα contained 68% iMet and 25% oxidized iMet, and 0.7% peptides that lacked iMet. Similarly, the *Se*HUβ contained 59% iMet and 37% oxidized iMet, and 4% of the sub-population lacked iMet. Given that in both cases, the sub-population of peptides that lacked iMet was so low, we hypothesized that such a population of peptides could have been generated in vivo or during the sample ionization.

### 3.4. SeNatB Did Not Acetylate N-Terminal Methionine Sulfoxide Residues

**Figure 4:**
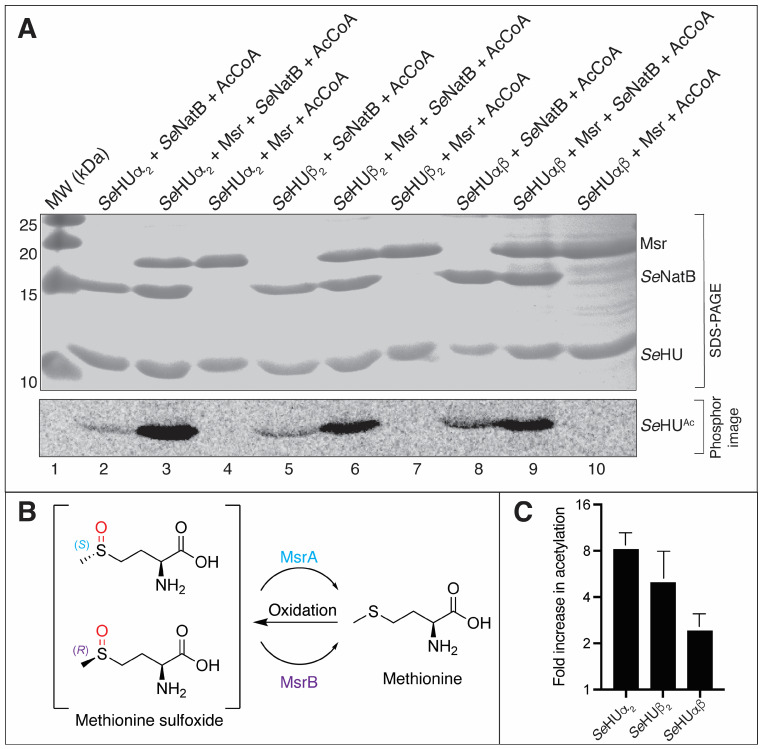
The reduction of Met-SO by MsrA and MsrB enzymes increased the *Se*NatB-mediated acetylation. (**A**) Radiolabeled acetylation assays were conducted via the incubation of an *Se*HUα_2_, *Se*HUβ_2_, or *Se*HUαβ protein (5 μM each) for one hour at 37 °C with (lanes 3, 6, and 9) or without (lanes 2, 5, and 8) 5 μM of MsrA and MsrB and 20 mM DTT. After an hour, 40 μM [acetyl-1-^14^C]-AcCoA and *Se*NatB (2 μM) were added and the reactions incubated for one additional hour. The negative control lanes 4, 7, and 10 included *Se*HU proteins with Msr and AcCoA to show that Msr could not transfer the radiolabel to *Se*HU. The reactions were resolved by SDS-PAGE and imaged as described for other acetylation assays. (**B**) Schematic showing the activities of Msr proteins. (**C**) Densitometry analysis of the fold increase in the radiolabel when the Msr protein was added to the acetylation reaction. The area of pixels was calculated in ImageJ for duplicate experiments, the fold change was calculated in Microsoft Excel v 16.98, and the fold increase in acetylation in the Msr-included lanes was plotted with a log_2_ *y*-axis in Prism GraphPad version 10. This experiment was repeated three independent times.

Because of the difficulty in detecting *N-*terminally acetylated *Se*HU proteins, we asked whether *Se*NatB could acetylate *N*-terminal methionines (iMets) that had been oxidized to methionine sulfoxides (iMet-SOs). In *S.* Typhimurium, the peptide-bound Met-SO was reduced to Met by methionine sulfoxide reductases (MsrA, MsrB). When Met was oxidized to Met-SO, two enantiomers were generated, namely, (*S*)-Met-SO and (*R*)-Met-SO (Figure S5A and Figure 4B). To determine whether the iMet-SO residue reduction led to increased acetylation by *Se*NatB, we purified the *Salmonella* MsrA and MsrB proteins and pre-incubated a mixture of MsrA and MsrB with the *Se*HU proteins and DTT dithiothreitol (DTT, Figure S5B) to reduce both the enantiomers of iMet-SO to iMet. After the iMet-SO reduction, we performed radiolabeling acetylation assays in the presence of *Se*NatB. Msr proteins require a reducing agent, such as thioredoxin or NAD(P)H, to reduce methionine sulfoxide, but DTT is commonly used in lieu of thioredoxin [48]. As shown in Figure 4 (lanes 3, 6, and 9), the inclusion of Msr enzymes in the reaction mixture resulted in an increased labeling of *Se*HU proteins as compared with *Se*NatB only (lanes 2, 5, and 8). The densitometry analysis of radiolabeled bands from reactions of *Se*HU proteins, AcCoA, and *Se*NatB with and without the Msr addition showed a 3–8-fold increase in acetylation of the HU proteins by the addition of Msr (Figure 4C). These results suggest that *Se*NatB acetylated the *N*-termini with iMet residues after their reduction from iMet-SO. Importantly, these data also demonstrate that the Msr proteins used *Se*HU proteins as substrates.

### 3.5. SeNatB and BsYfmK Acetylated Lysine-Null (K-Null) Variants of SeHU and HBsu Proteins In Vitro

Carabetta et al. showed that *Bs*YfmK acetylates seven lysyl groups of the *B. subtilis* HBsu protein [28].

**Figure 5:**
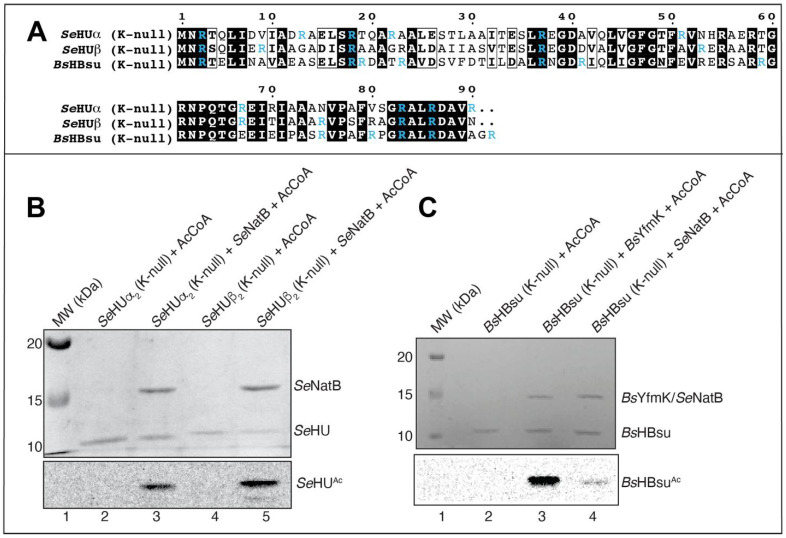
*Se*NatB and *Bs*YfmK acetylated the K-null variants of *Se*HU and HBsu, respectively. (**A**) Amino acid alignment of the HU proteins. Lysine residues that were changed to arginine are shown in blue color in all proteins. Identical residues are shown with a black background, while similar residues are boxed with a white background; dots represent residues present in the *B. subtilis* HBsu protein, but absent in the *S.* Typhimurium proteins. The figure was made using the ESPript 3.0 software package. (**B**) To verify the N-terminus as the site of acetylation, the K-null variants of *Se*HUα_2_ (HUα_2_^K3R,K13R,K18R,K22R,K37R,K51R,K67R,K83R,K86R,K90R^) and *Se*HUβ_2_ (HUβ_2_^K3R,K9R,K18R,K37R,K53R,K67R,K75R,K83R,K86R^) were purified and tested as substrates of *Se*NatB and [acetyl-1-^14^C]-AcCoA for acetylation. This experiment was conducted as described in the legend to Figure 2, where proteins were incubated with [acetyl-1-^14^C]-AcCoA in the presence or absence of *Se*NatB. (**C**) To verify that *Bs*YfmK could acetylate HBsu in the absence of lysyl residues, the K-null variant of HBsu (HBsu^K3R,K18R,K19R,K37R,K41R,K59R,K75R,K80R,K83R,K86R,K93R^) was purified and tested as a substrate for *Bs*YfmK and *Se*NatB using [acetyl-1-^14^C]-AcCoA. The bands seen on the phosphor image indicate acetylated protein that corresponded to protein bands from SDS-PAGE seen at the size of HU proteins, namely, 10 kDa. This experiment was conducted as described in the legend to Figure 2 and in Section 2. These experiments were repeated three independent times.

Figure S16 of the SI Appendix of the Carabetta et al. paper shows that mass spectrometry analysis does not unambiguously establish whether residue K3 or the *N*-terminus was acetylated based on protease peptide sequence coverage. Based on our knowledge of *Se*NatB function as an *N^α^*-acetyltransferase [24], we hypothesized that the *N*-terminus of HBsu was an additional site of enzymatic acetylation by *Bs*YfmK. To confirm that *Se*NatB acetylated the *N-*terminal methionine of *Se*HU, we generated K-null variants of *Se*HUα_2_ and *Se*HUβ_2_ by changing every K to R (Figure 5A). Both *Se*HUα_2_(K-null) and *Se*HUβ_2_(K-null) proteins were acetylated in vitro using homogenous *Se*NatB. As shown in Figure 5B (lanes 3, 5), the K-null variants of *Se*HUα_2_ and *Se*HUβ_2_ were strongly acetylated by *Se*NatB, indicating that both *Se*HU homodimers were substrates of *Se*NatB and that *Se*NatB acetylated the *N* terminus of each HU protein. These results were consistent with the reported *N^α^* acetyltransferase activity of *Se*NatB [24]. We also investigated whether *Bs*YfmK acetylated the *N*-terminus of *HBsu*. For this purpose, we purified the K-null variant of this protein (Figure 5A) and determined that *Bs*YfmK in fact acetylated HBsu in vitro (Figure 5C, lane 3). Notably, *Se*NatB also acetylated the HBsu K-null variant, albeit weakly (Figure 5C, lane 4).

**Figure 6:**
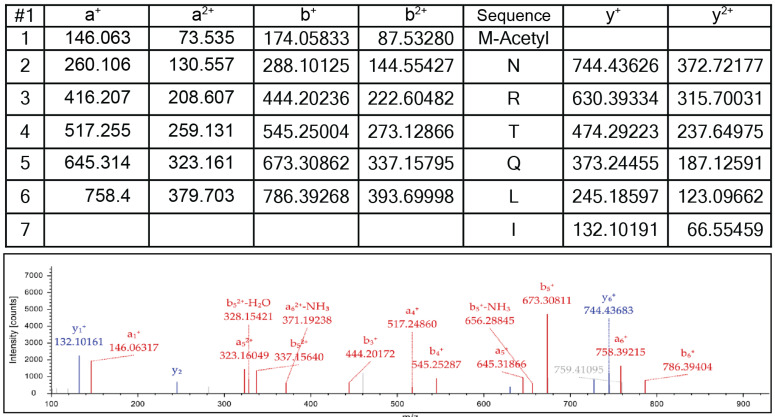
Mass spectra of the *N*-terminus of the *Se*HUa K-null variant of *Se*HU acetylated by *Se*NatB. K-null *Se*HUα protein (5 μM) was incubated with AcCoA (1 mM) and *Se*HUα (3 μM) at 37 °C for 1 h. The reaction mixture was resolved by 15% SDS-PAGE [41], and *Se*HUα was excised from the gel and digested by Asp-N following the protocol described under Section 2. The mass spectrum (lower panel) of Asp-N-digested K-null variant shows that the b ions were the series of fragments that extended from the *N* terminus; the y ions were the series of fragments that extended from the *C* terminus. The x-axis of *m*/*z* stands for mass (*m*) over the charge number of ions (*z*). MASCOT software (http://www.matrixscience.com, 11 November 2023) was the online search engine used to identify peptides based on their masses. This analysis was performed at the Proteomics and Mass Spectrometry (PAMS) facility of the University of Georgia.

The results of the LC-MS/MS analysis of the *Se*HUa K-null variant digested with Asp-N protease confirmed that the NatB acetyltransferase modified the *N*-terminal methionine (Figure 6).

**Figure 7:**
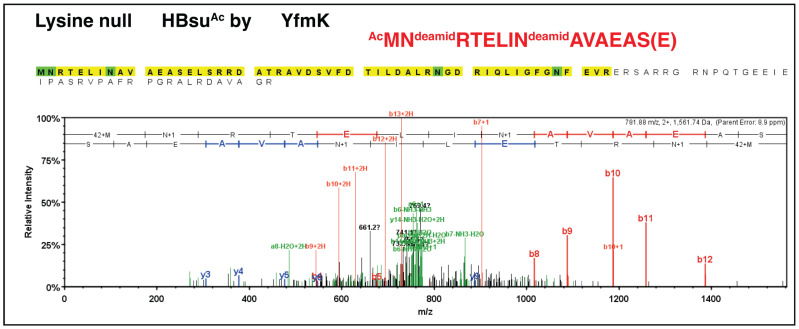
*Bs*Yfmk acetylated the *N*-terminus of the HBsu K-null variant of *B. subtilis.* The details of the protocol used for the analysis of the HBsu K-null variant after treatment with Asp-N protease are described under Section 2. The mass spectrum of the Asp-N-digested K-null variant shows that the b ions were the series of fragments that extended from the *N* terminus; the y ions were the series of fragments that extended from the *C* terminus. The *x*-axis of *m*/*z* stands for mass (m) over the charge number of ions (z). MASCOT software (http://www.matrixscience.com) was the online search engine used to identify the peptides based on their masses. This analysis was performed at the Biotechnology Center of the University of Wisconsin-Madison. Asparagine (N) residues shown in green were found deamidated.

An equivalent experiment was performed using the K-null HBsu variant and the *Bs*Yfmk enzyme from *B. subtilis.* As shown in Figure 7, *Bs*Yfmk modified the *N-*terminus of the K-null HBsu variant, indicating that *Bs*Yfmk displayed *Na*-acetylating activity in addition to its bona fide *N^e^*-lysine acetyltransferase activity [28].

### 3.6. N-Terminally Acetylated SeHU Proteins Could Not Be Deacetylated by the S. Typhimurium CobB Sirtuin Deacylase

**Figure 8:**
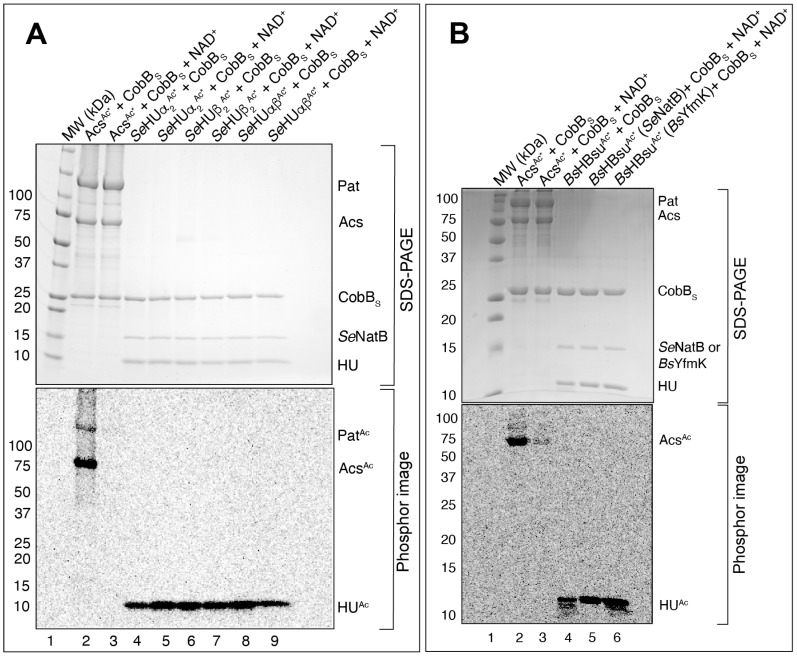
The CobB sirtuin deacylase could not deacetylate the acetylated *Se*HU proteins nor the acetylated HBsu protein. (**A**) [Acetyl-1-^14^C]-labeled *Se*HU proteins were incubated with NAD^+^ with CobB_S_ (lanes 5, 7, and 9) and without CobB_S_ (lanes 4, 6, and 8). A positive control of CobB deacetylating [acetyl-1-^14^C]-Acs (lanes 2 and 3) was included. (**B**) [Acetyl-1-^14^C]-labeled HBsu protein was incubated with NAD^+^ with CobB_S_ (lanes 5 and 6). Positive controls of CobB deacetylating [acetyl-1-^14^C]-Acs (lanes 2 and 3) were included. The bands seen on the phosphor image indicate acetylated protein that corresponded to protein bands from the SDS-PAGE seen at the size of HU proteins, namely, 10 kDa. The positive control acetylated Acs protein corresponded to a band seen on the phosphor image and SDS-PAGE at 75 kDa. The samples were resolved by SDS-PAGE and radiolabel transfer was visualized by phosphor imaging analysis. These experiments were repeated three independent times.

A feature of *N^α^* acetylation is that it is not reversible. To support the conclusion that *Se*NatB and *Bs*YfmK acetylated the *N*-termini of *Se*HU proteins, we acetylated *Se*HU proteins with [1-^14^C]-AcCoA and used them as substrates for the *S.* Typhimurium CobB deacylase short isoform [49]. The *Se*HUβ^Ac*^, *Se*HUα^Ac*^, and *Se*HUαβ^Ac*^ proteins acetylated by *Se*NatB, and the HBsu^Ac*^ protein acetylated by *Se*NatB or *Bs*YfmK were incubated with the short isoform of the CobB sirtuin deacylase (CobB_S_) in the presence or absence of NAD^+^. To ensure that the CobB_S_ protein was functional, CobB_S_ was incubated with radiolabeled Acs (Acs^Ac*^), a bona fide substrate of CobB_S_, in the presence or absence of NAD^+^. As shown in Figure 8A,B, CobB_S_ deacetylated Acs^Ac*^ (lane 3), but did not deacetylate *Se*HUβ^Ac*^, *Se*HUα^Ac*^, *Se*HUαβ^Ac^ (Figure 8A, lanes 5, 7, 9), nor HBsu^Ac*^ (Figure 8B, lane 6).

### 3.7. N-Terminal Acetylation of SeHU Proteins Altered SeHU-Dependent Gene Regulation

**Figure 9:**
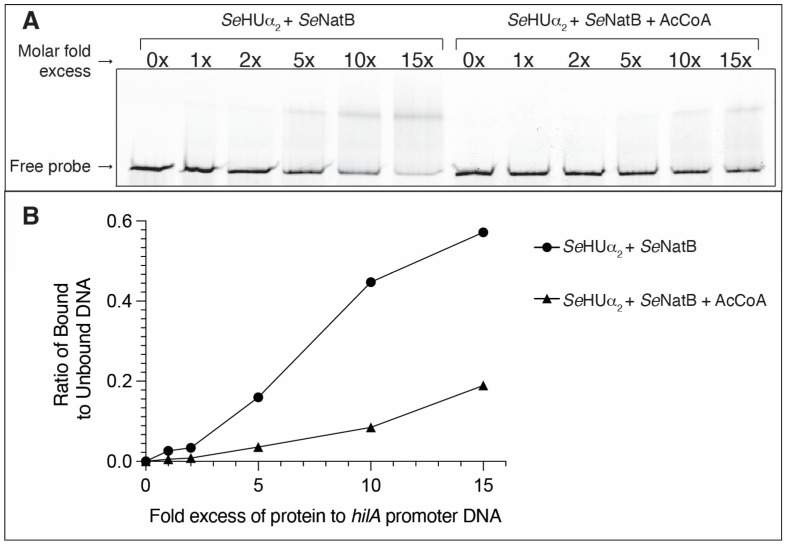
The *N*-terminal acetylation of *Se*HUα_2_ affected its DNA binding ability in vitro. (**A**) *Se*HUα_2_ was either pre-acetylated with *Se*NatB and cold acetyl-CoA or incubated with *Se*NatB and no AcCoA in a HEPES buffer. The *Se*HUα_2_^Ac^ or *Se*HUα_2_ proteins were added at increasing molar fold excess of protein to the dsDNA *hilA* probe and incubated at room temperature for 10 min. The reactions were resolved on a 7.5% TBE gel and imaged using a Typhoon Trio^+^ imager at a wavelength of 488 nm. This experiment was repeated three independent times. (**B**) Densitometric quantification of the ratio of DNA shifted when bound by *Se*HUα_2_ or *Se*HUα_2_^Ac^ as compared with free DNA in each lane of panel 9A. The measurements were made using ImageJ software. The ratios were calculated in Microsoft Excel, and the ratios were plotted using Prism 10 software.

After establishing that *Se*NatB acetylated *Se*HUβ_2_, *Se*HUα_2_, and *Se*HUαβ, we sought to determine the effect of acetylation on the *Se*HU-dependent regulation of gene expression in vitro and in vivo. To answer this question in vitro, we performed electrophoretic mobility shift assays (EMSAs) to quantify the changes in DNA-binding of acetylated HU proteins to bona fide DNA substrates. The *Se*HUα_2_ and *Se*HUβ_2_ proteins were pre-acetylated by *Se*NatB with AcCoA and incubated with a 5′ 6-FAM-labeled *hilA* promoter DNA probe. The *hilA* promoter was chosen as a DNA probe for *Se*HUα_2_ protein binding because it was shown that *Se*HU modulates the expression of the *hilA* gene in *S.* Typhimurium [50]. As shown in Figure 9A, the acetylated *Se*HUα_2_ did not bind as robustly at 10- or 15-fold molar excess of protein to the probe as the *Se*HUα_2_ protein pre-incubated with *Se*NatB in the absence of AcCoA. These results suggest that the *Se*HUα_2_ protein was acetylated by *Se*NatB, and that the acetyl moiety negatively affected the ability of *Se*HUα_2_ to bind to the *hilA* promoter. Densitometry analysis was performed on the gel shift bands to quantify the difference in the ability of *Se*HUα_2_ and *Se*HUα_2_^Ac^ to bind to the *hilA* promoter, where unacetylated *Se*HUα_2_ bound over 4, 5, and 3 times more protein to DNA at 5, 10, and 15-fold excess of HU to DNA as compared with acetylated *Se*HUα_2_ protein (Figure 9B).

#### In Vivo Evidence

**Figure 10:**
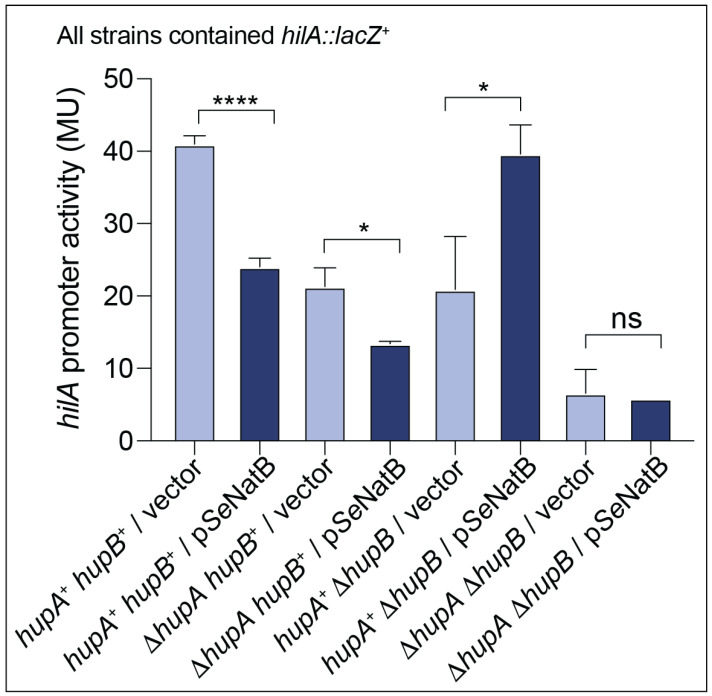
*Se*NatB affected the HU-dependent *hilA* gene expression. β-Galactosidase assays were used to assess the expression of the *lacZ* gene under the control of the *hilA* promoter as a function of *Se*NatB. The β-Galactosidase activity was quantified in cells grown under SPI-1 inducing conditions of low oxygen, at 37 °C, and in LB medium containing L-(+)-arabinose (0.5 mM) without shaking. The cultures were grown to mid-log phase, at which point the β-galactosidase activity was measured. The plasmid designation of the vector stands for pCV1 (empty plasmid) and p*Se*NatB stands for pNatB10 (encoding *natB* gene from *S. enterica*). Error bars indicate the standard deviation (SD). Strains were grown in biological duplicate and the experiment was conducted three independent times; ****, *p* < 0.0001; *, *p* < 0.0286; ns, not significant (two-tailed Student’s *t*-test assuming unequal variance); MU, Miller units.

It has been established that *Se*HU positively affects the transcriptional activation of *hilA*, which encodes the master regulator of invasion genes and the *Salmonella* Pathogenicity Island-1 (SPI-1) [50]. To test whether *Se*NatB altered the *Se*HU-mediated *hilA* expression in vivo, we monitored the expression of a chromosomal *hilA*::*lacZ*^+^ transcriptional fusion as a function of the ectopic expression of *Se*NatB. As expected, in a *hupB*^+^ *hupA*^+^ strain grown under conditions that triggered the expression of SPI-1 (i.e., high salt, low oxygen), *Se*HU positively affected the *hilA* expression [33,50]. However, when *Se*NatB was ectopically expressed, the level of expression of the *hilA*::*lacZ*^+^ fusion decreased ~40% (Figure 10).

When the *hupA* gene was deleted, the *hilA* expression decreased to levels seen in the *hupA*^+^ *hupB*^+^ strain that expressed *Se*NatB, and when *Se*NatB was overexpressed in the Δ*hupA* strain, the *hilA* expression level further decreased, suggesting a decrease in the activation of *hilA* by *Se*HUβ_2_ in the presence of *Se*NatB. When *hupB* was deleted, the *hilA* expression dropped to levels that mimicked those observed in the Δ*hupA* strain or the *hupA*^+^ *hupB*^+^ strain that ectopically expressed *S.* Typhimurium *natB*^+^. However, when *Se*NatB was overproduced in a Δ*hupB* strain where *Se*HUα_2_ was the only HU protein available to induce *hilA* expression, we unexpectedly saw an increase in the expression of *hilA.* This result was unanticipated, especially since Figure 7 shows that acetylated *Se*HUα_2_ protein displayed a decreased ability to bind to the *hilA* promoter in vitro. Additional work is needed to better understand this result. Finally, the *hilA* expression in a Δ*hupA* Δ*hupB* strain was not different than the *hilA* expression in a Δ*hupA* Δ*hupB* strain in which *Se*NatB was overproduced, suggesting that *Se*NatB cannot alter *hilA* expression in the absence of the *hup* genes under the condition tested (Figure 10).

## 4. Discussion

### 4.1. Is the N^α^ Acetylation of HU Proteins a Response to Oxidative Damage?

The finding that *Se*HU proteins undergo multiple *N*-terminal modifications, including iMet excision, methionine oxidation, and *N*-terminal acetylation is novel and sheds light on the importance of *N*-terminal modification in the modulation of the function of this important nucleoid-associated protein. At first, the finding that *Se*HU proteins have iMet cleavage was surprising due to results of in vitro analyses using the methionine aminopeptidase (MetAP) enzyme. The alluded data suggest that when the position 2 (P2) residue was asparagine (Asn), those proteins were poor substrates for MetAP. However, further analysis into *N*-terminomics in bacteria revealed that the in vitro studies of MetAP activity did not faithfully reflect in vivo iMet cleavage [22,51], whereas bioinformatics analyses across several species of bacteria showed that the cleavage of iMet with Asn as the P2 residue is a common occurrence and physiologically relevant [52].

**Figure 11:**
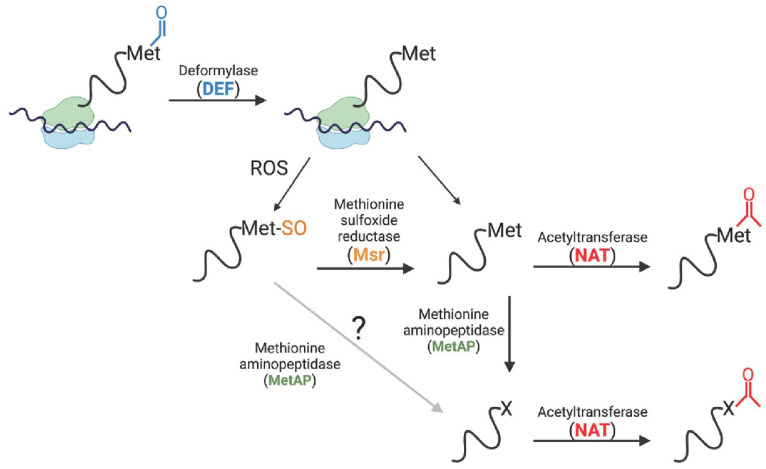
Model of possible HU protein *N*-termini fates in *S.* Typhimurium. Based on recent bacterial protein *N*-terminomics data and the findings reported herein, a summary of the different fates of HU proteins *N*-termini is as follows; proteins are deformylated by DEF deformylase, where the initiator methionine can be either oxidized to methionine sulfoxide or acetylated by NATs, such as *Se*NatB, in the *N*-terminal amino group. Alternatively, the iMet residue can be excised by methionine aminopeptidase (MetAP). Some proteins can be acetylated on the remaining *N*-terminal amino group (represented by an X in the figure). It is not known whether MetAP can excise *N*-terminal methionine sulfoxides in vivo in bacteria, so a question mark has been placed in this area of the schematic.

Our data show that *Se*HU proteins that retain their iMet are susceptible to *N*-terminal methionine oxidation to methionine sulfoxide (Figures S3 and S4), an oxidation that can be reversed by the methionine sulfoxide repair enzymes in *S.* Typhimurium (Figure 4 and Figure S5). This work also demonstrated that iMet-SO *N*-termini were poor substrates for *Se*NatB, and the repair of iMet-SO to iMet restored *Se*NatB-mediated *N^α^* acetylation (Figure 4). Perhaps the Msr-dependent repair of iMet oxidation is what triggers *Se*NatB-dependent *N^α^* acetylation, which could suggest a role for acetylated iMet *N*-termini in the cellular response to oxidative damage. In Figure 11, we present a scheme showing the different possibilities of *N*-terminal protein processing we observed during this work. Our results suggest that proteins with acetylated iMet or P2 residues appear to be stable proteins, but additional work is needed to monitor the protein stability as a function of the *N^α^* acetylation state. This scheme, however, is consistent with the results from recent bacterial protein *N*-terminomics data obtained by others [52,53,54].

From our previous study of *N^α^* acetylation of CobB_L_ by *Se*NatB, we found that *Se*NatB can accommodate up to two extra residues on the *N*-terminus of CobB_L_ and still recognize CobB_L_ as an acetylatable substrate [24]. However, at present, it is unclear whether *Se*NatB can acetylate *Se*HU lacking the iMet residue, e.g., *Se*HU proteins whose *N*-terminal residue is Asn. Further analysis of *Se*NatB substrate recognition is needed.

### 4.2. Our Work Revealed Unknown Specificities of the B. subtilis YfmK Acyltransferase

Recent studies showed that the YfmK protein of *B. subtilis* (*Bs*Yfmk) acetylates several epsilon amino groups of lysines of the HBsu protein of this bacterium [28]. Importantly, in the alluded publication, the authors did not rule out the *N^α^* acetylation of HBsu by *Bs*YfmK. However, the authors convincingly demonstrated that *Bs*YfmK had *N^ε^* acetyltransferase activity. The results reported here show that *Bs*YfmK also has *N^α^* acetyltransferase activity (Figure 5C). More work is needed to understand the fate of *N^α^* acetylated proteins and to identify the interplay of the *N^α^* and *N^ε^* acetytransferase activities of *Se*NatB and *Bs*YfmK and the impact of these activities on the metabolisms of *S.* Typhimurium and *B. subtilis.*

## Figures and Tables

**Table 1:** Strains used in this study ^1^.

Strain	Relevant Genotype	Reference/Source
***S. enterica* strains**		
JE6583 ^2^	*metE205* Δ*araB9*	Kenneth Sanderson
JE6692	*metE205* Δ*araB9/*pKD46	
JE9637 ^3^	*aadA*::*araCPBADT7-1*	[32]
JE13152	*aadA*::*araCPBADT7-1* Δ*hupA102* Δ*hupB104 lon-71 zaj-1034*::Tn*10 pat*::MudI1734	
JE7658 ^4^	*S. enterica* serovar Typhimurium 14028s (wild-type genome)	Joshua Fierer
**Derivatives of JE7658**		
JE18227	*att*λ::pDX1::*hilA*′-*lac*^+^	[33]
JE25581	*att*λ::pDX1::*hilA*′-*lac*^+^ *hupA101*::*kan*^+^/pCV1	
JE25582	*att*λ::pDX1::*hilA*′-*lac*^+^ *hupA101*::*kan*^+^/pNatB10	
JE25583	*att*λ::pDX1::*hilA*′-*lac*^+^ *hupB103*::*cat*^+^/pCV1	
JE25584	*att*λ::pDX1::*hilA*′-*lac*^+^ *hupB103*::*cat*^+^/pNatB10	
JE25588	*att*λ::pDX1::*hilA*′-*lac*^+^ *hupB103*::cat^+^ *hupA101*::*kan*^+^/pCV1	
JE25589	*att*λ::pDX1::*hilA*′-*lac*^+^ *hupB103*::*cat*^+^ *hupA101*::*kan*^+^/pNatB10	
***E. coli* Strains**		
*E. coli* C41 (λDE3)	*pka12*::*kan*^+^ *ompT hsdS* (r_B_m_B_) *gal* (λDE3)	Laboratory collection
*E. coli* C43 (λDE3)	*F-ompT gal hsdSB* (r_B_-m_B_-) *dcm lon* C41 (λDE3)	Laboratory collection

^1^ If no reference or source is indicated, it means the strain was constructed during this work. ^2^ *Salmonella enterica* subsp. *enterica* sv. Typhimurium str. LT2. ^3^ *Salmonella enterica* subsp. *enterica* sv. Typhimurium str. SB300. ^4^ *Salmonella enterica* subsp. *enterica* sv. Typhimurium 14028s.

**Table 2:** Plasmids used in this study ^1^.

Plasmid	Genotype	Description	Source
pCV1	*araC* ^+^ *bla* ^+^	P*_araBAD_* expression vector	[36]
pNatB10	*natB* ^+^ *bla* ^+^	*S. enterica natB*^+^ cloned into pCV1	[24]
pTEV6	*bla* ^+^	N-terminal rTEV-cleavable MBP-His fusion overexpression vector	[37]
pYfmk1	*yfmK* ^+^ *bla* ^+^	*B. subtilis yfmK*^+^ cloned into pTEV6	
pNatB6	*natB* ^+^ *bla* ^+^	*S. enterica natB*^+^ cloned into pTEV6	[24]
pTEV18	*bla* ^+^	N-terminal rTEV-cleavable His fusion overexpression vector	[36]
pMsrA2	*msrA*+ *bla*^+^	*(Stm4408) msrA* cloned into pTEV18	
pMsrB1	*msrB* ^+^ *bla* ^+^	*stm1291* (*yeaA* ^+^ also called *msrB*) cloned into pTEV18	
pT7-7	*bla* ^+^	Overexpression vector with T7 promoter	[38]
pSAPKO-WT	*kan* ^+^	Derivative of pET28b with BspQI cut sites and T7 promoter for overexpression of tagless vectors	[39]
pHBSU1	*hbs* ^+^ *kan* ^+^	*hbs*^+^ cloned into pSAPKO-WT	
pHUPB3	*hupB* ^+^ *bla* ^+^	Encodes HupA^WT^ in pT7-7	
pHUPA5	*hupA* ^+^ *bla* ^+^	*hupB*^+^ cloned into pT7-7 (Contains extra start ATG)	
pHUPA6	*hupA* ^+^ *bla* ^+^	Encodes HupA^K3R,K13R,K18R,K22R,K37R,K51R,K67R,K83R,K86R,K90R^ in pT7-7	
pHUPA7	*hupA* ^+^ *bla* ^+^	Encodes HupB^WT^ with deleted extra ATG in pT7-7; mutagenized pHUPA3	
pHUPB14	*hupA* ^+^ *bla* ^+^	Encodes HupB^K3R,K9R,K18R,K37R,K53R,K67R,K75R,K83R,K86R^ in pT7-7	
pHUPB15	*hupB* ^+^ *bla* ^+^	Encodes HupB^WT^ with deleted extra ATG in pT7-7; mutagenized pHUPB5	
pKD46	*exo* ^+^ *bet* ^+^ *gam* ^+^ *bla* ^+^	Expression of lambda Red recombinase system	[24]

^1^ Plasmids were constructed during this work unless otherwise indicated.

**Table 3:** Primers used in this study ^1^.

Primer Name	Primer Sequence 5′ → 3′
pYiaC10_F_BspQI	nngctcttcnttcatgattcgcaaatcccagagtgaagac
pYiaC10_R_BspQI	nngctcttcnttattacggcgtttgatccgcctgccaac
msrB_F_BspQI	nngctcttcnttcatgagcacgtttaaagtgag
msrB_R_BspQI	nngctcttcnttatcagcctttcagttgat
Yfmk_F_BspQI	nngctcttcnttcatggcttcaatagacagg
YfmK_R_BspQI	nngctcttcnttatcagttgcgaagaatcag
HBsu_F_sapkowT	nngctcttcnatgatgaacaaaacagaact
HBsu_R_sapkowT	nngctcttcnttaagttgccggaaaataa
Del_M1_HupA1	ctttaagaaggagatatacatatgaacaagactcaactgattgatgta
Del_M1_HupA2	tacatcaatcagttgagtcttgttcatatgtatatctccttcttaaag
DelM1_HupB1	tttaactttaagaaggagatatacatatgaataaatctcaactgatcgaaaaaattgc
DelM1_HupB2	gcaattttttcgatcagttgagatttattcatatgtatatctccttcttaaagttaaa
SeHupB_K3R_P1T77	tttcgatcagttgagatctattcaccatatgtatatctccttcttaaagt
SeHupB_K3R_P2T77	actttaagaaggagatatacatatggtgaatagatctcaactgatcgaaa
SeHupB_K9R_P1	cagcccctgcagcaattctttcgatcagttgagat
SeHupB_K9R_P2	atctcaactgatcgaaagaattgctgcaggggctg
QC1_K18R_HupB	caggggctgatatctctagggctgcggctg
QC2_K18R_HupB	cagccgcagccctagagatatcagcccctgc
QC1_K37R_HupB	tcatccccttctctcagagattcggtaacagaagca
QC2_K37R_HupB	tgcttctgttaccgaatctctgagagaaggggatga
QC1_K75R_HupB	ctcggcactctggcagcggcgatggt
QC2_K75R_HupB	accatcgccgctgccagagtgccgag
QC1_ K83R_86R_HupB	taccgcgtctctcagcgctctacctgcacggaaactcg-
QC2_ K83R_86R_HupB	cgagtttccgtgcaggtagagcgctgagagacgcggta
hilA_R1_FAM	taaaatgtggcatgataatagt
hilA_F1	ctattgcaatgaggcca
yfmk_F_KpnI-pTEV6	nnnggtaccatggcttcaatagacagg
yfmk_R_NotI-pTEV6	nnngcggccgctcagttgcgaagaatcag

^1^ All primers were synthesized by Integrated DNA Technologies, Coralville, IA, USA. red color: highlight the change made.

## Data Availability

All the data generated during this work are contained in this article and its Supplementary Materials.

## Supplementary Materials

The following supporting information can be downloaded from https://www.mdpi.com/article/10.3390/pathogens14070616/s1: Figure S1: (A) *Se*NatA and *Bs*YfmK similarity and identity level. (B) *Se*HUα, *Se*HUβ, and HBsu amino acid sequence alignment. Figure S2: Asp-N endoproteinase digestion mapping of *Se*HU proteins. Figure S3: Extracted ion chromatograph (EIC) analysis of the *N*-terminus of SeHUα separated from the HU heterodimer. Figure S4: Extracted ion chromatograph (EIC) of the *N*-terminus of SeHUβ separated from HU heterodimer. Figure S5: Methionine sulfoxide reductases are active in vitro as shown by TLC.
