# Supplementary material for: In Salmonella Typhimurium and Bacillus subtilis, Nucleoid-Associated HU Proteins Are N-Terminally Acetylated
Source: Pathogens. 2025 Jun 20;14(7):616. doi: 10.3390/pathogens14070616 (PMC12299546; doi:10.3390/pathogens14070616)
Supplement: Supplementary file 1 [file pathogens-14-00616-s001.zip › pathogens-3642675-supplementary.pdf]

SUPPORTING INFORMATION

In *Salmonella* Typhimurium and *Bacillus subtilis* nucleoid-associated HU proteins are *N*-terminally acetylated.

Anastacia R. Parks[1§], Jessica L. Will[1], Liju, G. Mathew[1†], Sébastien Massier[2], Julie Hardouin[2,3], and Jorge C. Escalante-Semerena[1*].

[1]Department of Microbiology, University of Georgia, 330G Cedar Street Building C, 136 Cedar Street, Athens, GA 30602 USA.
[2]University of Rouen Normandie, INSERM US 51, CNRS UAR 2026, HeRacLeS-PISSARO, Normandie Université, 76000 Rouen, France
[3]University of Rouen Normandie, INSA Rouen Normandie, CNRS, Polymers, Biopolymers, Surfaces Laboratory UMR 6270, F-76000 Rouen, France

*Email: jcescala@uga.edu

[§]Current address: Department of Microbiology and Immunobiology, Harvard Medical School
[†]Current address: Meridian Life Science, Memphis Tennessee 38134
Keywords: *N*-terminal acetylation, HU protein, Gram-negative bacteria, Gram-positive bacteria, acetyltransferase, post-translational modifications, nucleoid compaction

This file includes:

Figures S1 to S5
Legends for figures S1 to S5

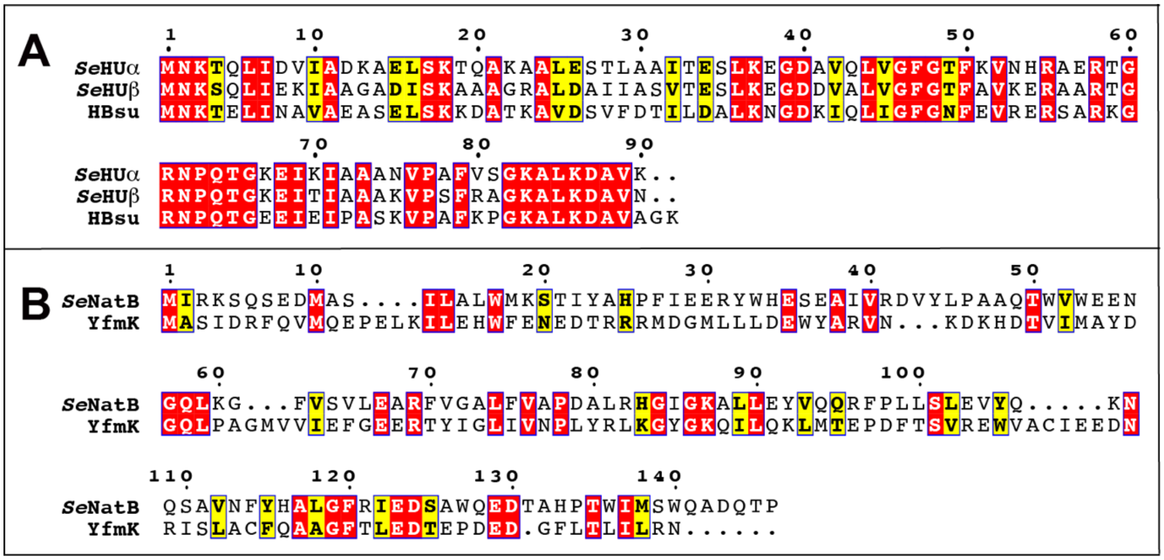

Fig. S1. A. *Se*NatA and *Bs*YfmK similarity and identity level. B. *Se*HUα, *Se*HUβ, and HBsu amino acid sequence alignment. Similar residues are written in black, boldtype face characters and boxed in yellow. Identical residues are boxed in red. Figure generated using ESPript 3.0 software.

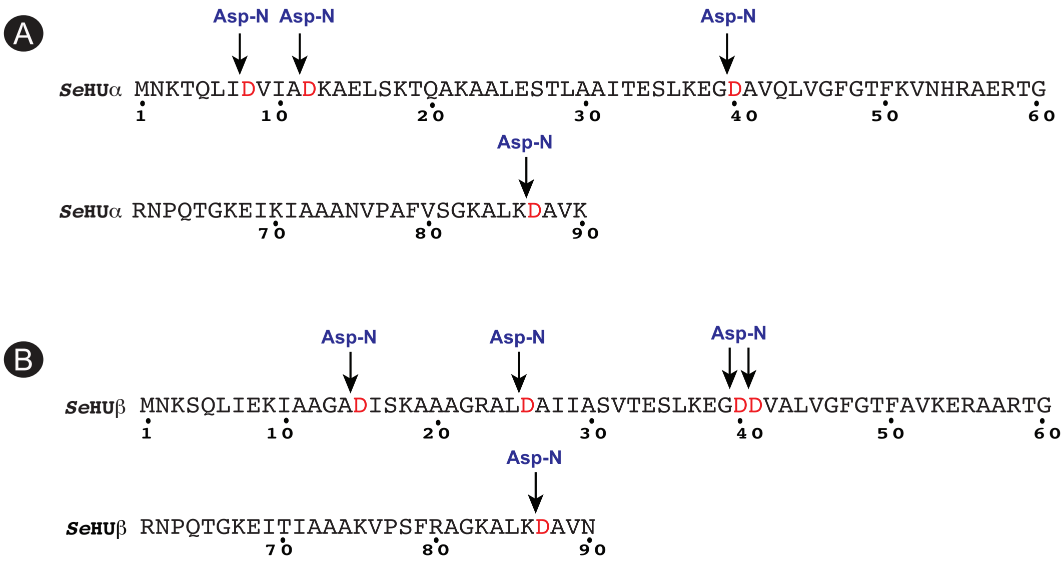

Fig. S2. Asp-N endoproteinase digestion mapping of *Se*HU proteins. The predicted digestion sites of A: *Se*HUα or panel B: *Se*HUβ by Asp-N protease, which cleaves N-terminal to aspartic acid residues, as labeled in red.

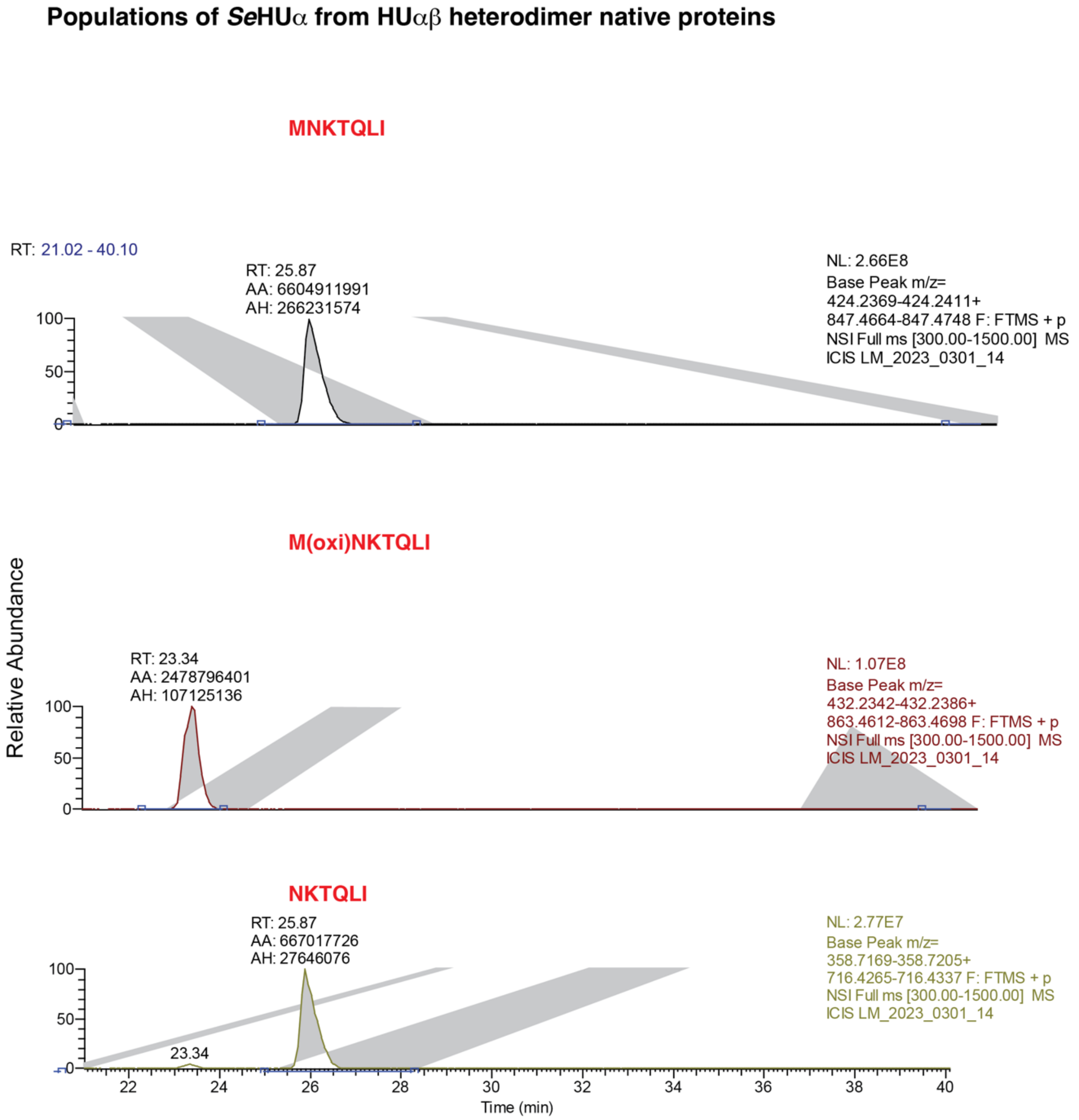

Figure S3. Extracted ion chromatograph (EIC) analysis of the N-terminus of *Se*HUα separated from the HU heterodimer. Purified, chromosomally-encoded HU heterodimer from *S. enterica* was resolved on a 7.5% TAU gel to separate the α and β subunits. The HUα protein was excised from the gel, digested with Asp-N protease and analyzed by LC-MS/MS.

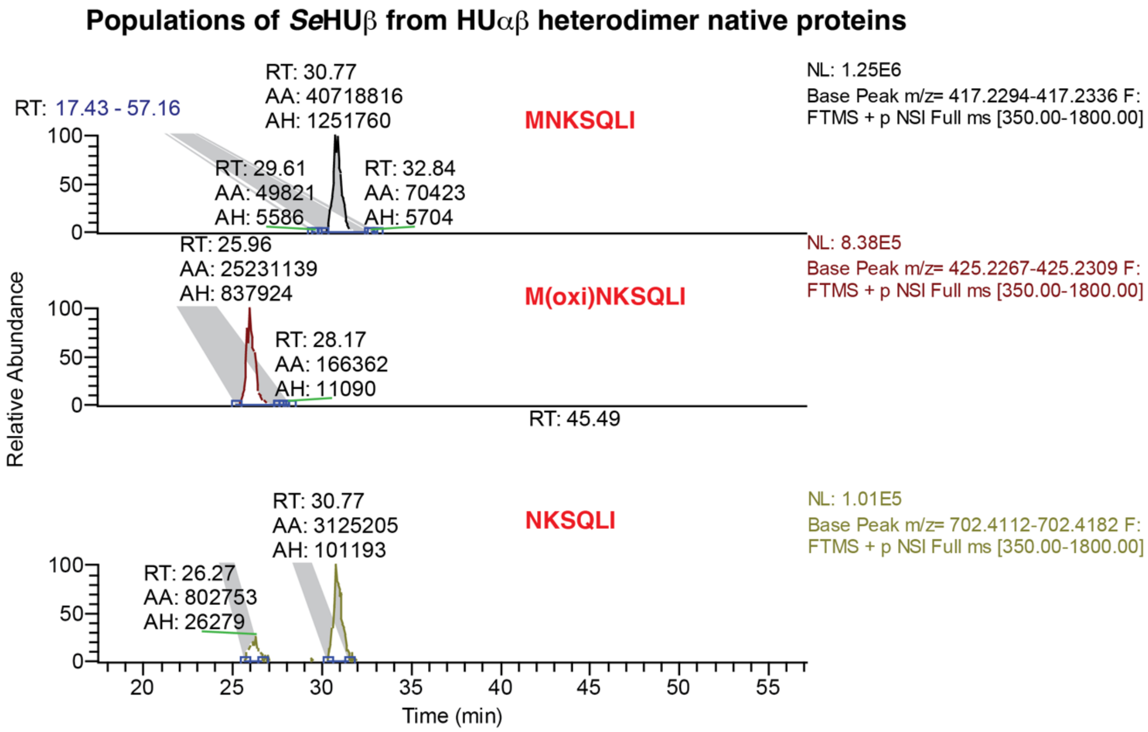

Figure S4. Extracted ion chromatograph (EIC) of the N-terminus of SeHUβ separated from HU heterodimer. Native-no-plasmid purification of HU heterodimer from *S.* Typhimurium was resolved on a 7.5% TAU gel to separate the α and β subunits. The HUβ protein was excised from the gel, digested with Asp-N protease and analyzed by LC-MS/MS.

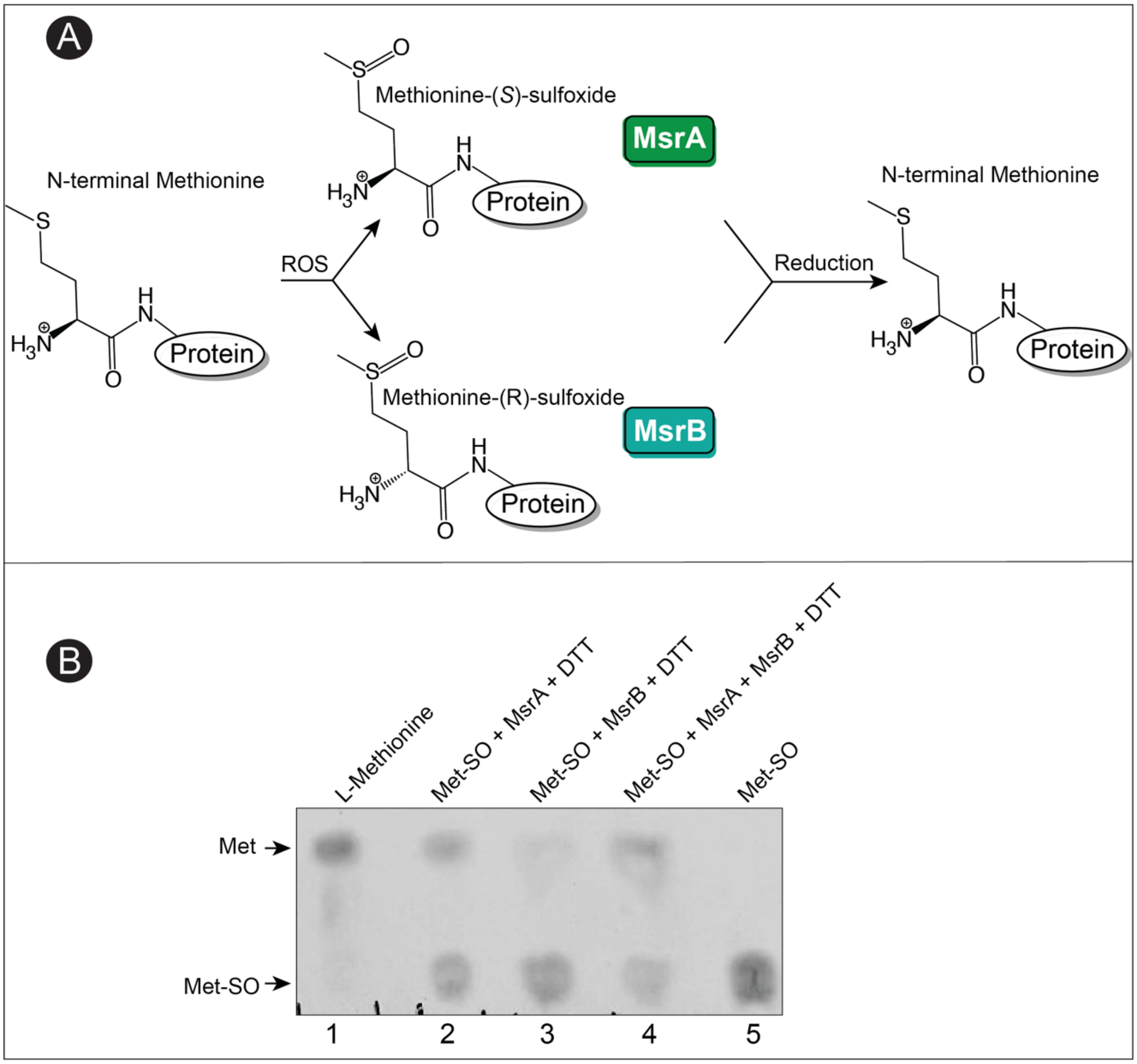

Figure S5. Methionine sulfoxide reductases repair stereospecific oxidized methionines of proteins. A. Schematic of how Msr enzymes repair methionine sulfoxide, but repair by Msr is diastereoselective. **B.** TLC verification of enzymatic activity of *Se*Msr proteins purified to homogeneity.
